# A Comprehensive Analysis on the Regulatory Network Underlying Callus Induction and Adventitious Organogenesis Process in Stem of *Populus Alba* L.

**DOI:** 10.3390/ijms26094087

**Published:** 2025-04-25

**Authors:** Xiao-Yuan Li, Gui-Feng Liu, Qing-Yin Zeng, Yan-Jing Liu

**Affiliations:** State Key Laboratory of Tree Genetics and Breeding, Northeast Forestry University and Chinese Academy of Forestry, Harbin 150040, China; lxiaoyuan@126.com (X.-Y.L.); liuguifeng@126.com (G.-F.L.); qingyinzeng@163.com (Q.-Y.Z.)

**Keywords:** *Populus alba*, endogenous hormones, transcriptional regulation, callus formation, adventitious shoot regeneration

## Abstract

*Populus* species are important resources for ecological conservation and certain industry productions, and are also considered model tree species for scientific research. For tree species, in vitro plant regeneration is an important method of propagation due to the advantage of high multiplication rate. Although many molecular determinants for poplar regeneration have been investigated, the complete regulatory hierarchy network remains unclear. In this study, we tracked the temporal changes of endogenous hormone contents, physiological characteristics and transcriptional profiles during callus induction and adventitious organogenesis in a stem of *Populus alba* L. to explore the regulatory dynamics of in vitro regeneration in poplars. The results imply that auxin may promote the formation of callus in *P. alba* by activating the expression of *WOX11/12*. By up-regulating the expression of *CUC1/2*, the development of callus begins to initiate apical meristem (SAM) at day 12. The cytokinin-mediated pathway regulates the adventitious shoot formation by *ESR1* and *WUS*. The precursors of active gibberellin GA1, GA53 and GA19 were accumulated in the early stage of callus induction, and then they continued to decrease. JA may function on adventitious shoot regeneration due to its accumulation after 12 days of induction. The dominant hormonal components and regulatory factors during regeneration were identified. Based on the results, a regeneration pathway regulated by auxin and cytokinin for poplars is proposed. The key regulators identified in this study will accelerate the exploration and understanding of the asexual reproduction mechanism of poplar trees.

## 1. Introduction

In vitro plant regeneration is an important method of plant propagation. During this process, plant cells form complete plants through dedifferentiation and redifferentiation [1,2,3]. During tissue culture, this process can be promoted by exogenous hormones [4]. It is found that auxin-induced callus formation represents a typical change in cell fate, where some somatic cells acquire pluripotency, and auxin and cytokinin play a key role in determining the reprogramming fate of cells [5]. Non-embryogenic callus with pluripotency is first induced on a callus-inducing medium (CIM) with high auxin and low cytokinin concentrations [6]. Then, the non-embryogenic callus is transferred to a shoot-inducing medium (SIM) with high cytokinin and low auxin concentrations to induce adventitious shoots for plant regeneration [7,8].

The molecular mechanism of cell fate change during organogenesis in *Arabidopsis thaliana* has been extensively studied. Auxin-induced callus formation shows characteristics of the root development [9,10]. *LATERAL ORGAN BOUNDARIES DOMAIN* (*LBD*) transcription factors *LBD16*, *LBD17*, *LBD18* and *LBD29* induced by auxin play a key role in callus formation [11,12,13]. They act on the downstream of *AUXIN RESPONSE FACTOR7* (*ARF7*) and *ARF19*, mediating lateral root emergence [14,15,16]. *PLETHORA3* (*PLT3*), *PLT5* and *PLT7*, which are essential for lateral root primordium development, can be induced by auxin on CIM. They activate the expression of *PLT1* and *PLT2*, which regulate the establishment of root meristem cell niche [17,18], thereby giving the callus the ability to form adventitious shoots. Thus, the genetic process that regulates lateral root formation is necessary not only for callus formation, but also for callus to acquire pluripotency [19]. In addition, wound response factor *WOUND-INDUCED DEDIFFERENTIATION* (*WIND*) can regulate callus formation by mediating the activation of ARABIDOPSIS RESPONSE REGULATOR (ARR)-dependent cytokinin signaling [20]. The formation of adventitious shoots requires the activation of genes associated with apical meristem (SAM) [21]. WUSCHEL (WUS) is a crucial regulator of shoot de novo regeneration and plays an important role in maintaining the activity of SAM stem cells [22,23]. Cytokinin induces adventitious organogenesis by mediating the transcription of *WUS* [24]. CUPSHAPED COTYLEDON1 (CUC1) and CUC2 are required for SAM initiation and are expressed in CIM-induced callus, which is significantly related to cell pluripotency [25]. Additional phytohormones that affect the process of shoot regeneration include brassinosteroids (BRs), gibberellins (GAs), and abscisic acid (ABA). BRs can promote de novo shoot organogenesis by regulating cell division and differentiation. The *DWARF4* gene, which encodes an enzyme that catalyzes a rate-determining step in BR biosynthetic pathways, is highly expressed in actively dividing calli [26]. Meanwhile, GAs suppress shoot regeneration. Exogenous application of GA reduces shoot formation, whereas treatment with paclobutrazol, a potent inhibitor of GA biosynthesis, increases the rate of shoot regeneration. Ethylene exerts both positive and negative impacts on shoot regeneration, depending on the sensitivity of explants to ethylene signaling [27].

In vitro regeneration of poplar was first introduced in 1934 [28]. Recently, lots of research on in vitro regeneration of poplar has been introduced. Research has shown that the vital transcriptional events in the regulation occur in the early stage of dedifferentiation. Poplar genes involved in auxin signaling are subject to complex regulation during regeneration. Most of the identified cell cycle genes are up-regulated during callus induction [29]. In addition, *WUSCHEL-RELATED HOMEOBOX11* (*WOX11*) is a key gene involved in auxin response and cell fate transformation [30]. *PtWOX11* has a regulatory effect on the regeneration of primary buds and initial roots of poplar. Moreover, a wound can induce the expression of *PtWOX11* and promote the formation of adventitious shoots from callus [31]. Although many dominant regulatory factors for poplar regeneration in vitro have been identified, the complete regulatory hierarchy network remains unclear. In this study, we tracked the temporal changes of endogenous hormone contents, physiological characteristics and transcriptional profiles during the callus induction and adventitious organogenesis process in a stem of *Populus alba* L. Based on these results, candidate key regulatory genes governing callus induction and adventitious shoot regeneration in *P. alba* were screened. Core functional components of endogenous hormonal networks were identified. These comprehensive data sets are expected to accelerate the elucidations of molecular regulatory mechanisms of poplar regeneration.

## 2. Results

### 2.1. Callus Induction and Adventitious Organogenesis of Populus alba

**Figure 1:**
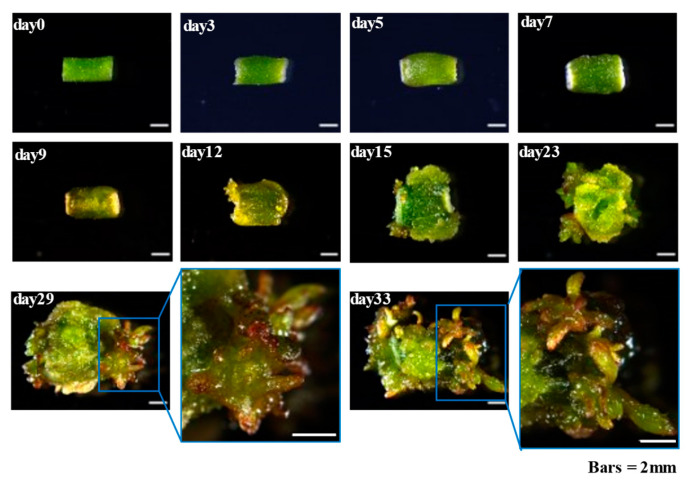
Callus induction and adventitious organogenesis in *P. alba*. The enlarged images of the adventitious shoot regeneration regions are shown on the right side of days 29 and 33.

In this study, internode stem segments of *P. alba* seedlings were used to induce callus and adventitious shoot. Through in vitro induction, the explant cells of *P. alba*. started to dedifferentiate and form callus within 3 days. On the third day of cultivation, slight swelling could be observed in the explants. Starting from the fifth day, the wound edges at both ends of the explant turned white and gradually swelled. On the seventh day, obvious callus tissue was observed at the wound site of the explant. Continuing cultivation for 12 days, the callus tissue gradually enlarged and adventitious shoots appeared. By day 15, it could be clearly observed that the callus continued to grow and the number of adventitious shoots significantly increased. Finally, after induction for 33 days, a large number of adventitious shoots were formed (Figure 1).

### 2.2. Dynamics of Endogenous Hormones During Callus Induction and Adventitious Organogenesis

#### 2.2.1. Auxins

**Figure 2:**
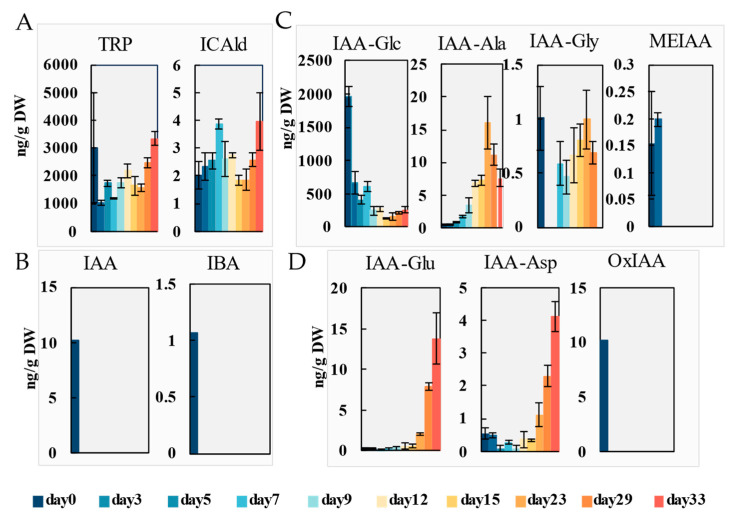
Dynamics of auxin precursors (**A**), active form (**B**), storage form (**C**) and metabolites (**D**) during callus induction and adventitious organogenesis. L-tryptophan (TRP), indole-3-carboxaldehyde (ICAld), indole-3-acetic acid (IAA), indole-3-butyric acid (IBA), IAA-glucose (IAA-Glc), IAA-alanine (IAA-Ala), IAA-glycine (IAA-Gly), methylindole-3-acetic acid (MEIAA), IAA-L-glutamate (IAA-Glu), IAA-L-aspartate (IAA-Asp), 2-oxindole-3-acetic acid (OxIAA). Data are shown as mean ± standard deviation (SD) for three independent biological replicates. The concentrations were determined with LC-MS/MS.

Auxins participate in the elongation and division of plant cells, playing a crucial role in plant regeneration. The endogenous auxin content was analyzed throughout the process. The results showed that a total of 11 auxin compounds (Trp, ICAld, IAA, IBA, IAA-Glc, IAA-Ala, IAA-Gly, MEIAA, IAA-Glu, IAA-Asp, OxIAA) were identified during the induction of callus and adventitious organogenesis (Figure 2). Among them, the accumulation level of L-tryptophan (Trp), a precursor for auxin synthesis, was the highest over the induction process. The endogenous Trp content measured at day 0 (immediately post-cutting) was significantly higher than that at day 3 (post-induction), indicating a rapid decline in Trp levels during the initial three days of treatment. Subsequently, from day 3 to day 33, Trp content exhibited a progressive upward trend. Indole-3-formaldehyde (ICAld), as another precursor of auxin, had a relatively low overall content, below 3.98 ng/g throughout the entire induction process. Indole-3-acetic acid (IAA) and indole-3-butyric acid (IBA), two active auxins, were only detected to accumulate at day 0 (hormone levels in stem segments immediately post-cutting), at 10.2 ng/g and 1.07 ng/g, respectively, and were not detectable during subsequent induction processes.

An ester-linked auxin IAA-glucose (IAA-Glc), a storage form of IAA, also accumulated significantly throughout the regeneration induction process. The highest content was 1963 ng/g at the beginning of callus induction. As callus and adventitious shoots formed, there was a temporary increase in the accumulation level of IAA-Glc between the fifth and seventh days. Then, it gradually decreased, reaching a minimum of 126 ng/g on day 15. Subsequently, with the growth of adventitious shoots, the accumulation level of IAA-Glc slowly increased again, reaching 258 ng/g on day 33. In contrast, the accumulation levels of methyl-linked auxin methylindole-3-acetic acid (MEIAA), ester-linked auxin IAA-alanine (IAA-Ala) and IAA-glycine (IAA-Gly) in storage form was lower than that of IAA-Glc.

IAA-L-glutamate (IAA-Glu) and IAA-L-aspartate (IAA-Asp) are amide-linked auxin degrading metabolites, which gradually accumulated on the 23rd day with the growth of adventitious shoots. On the 33rd day, the contents of IAA-Glu and IAA-Asp reached their highest levels, at 13.8 ng/g and 4.13 ng/g, respectively. At the initial stage of callus induction, a small accumulation of the oxidation product OxIAA of IAA was detected at a concentration of 10.2 ng/g, and was not detected at any other time points.

#### 2.2.2. Cytokinins

**Figure 3:**
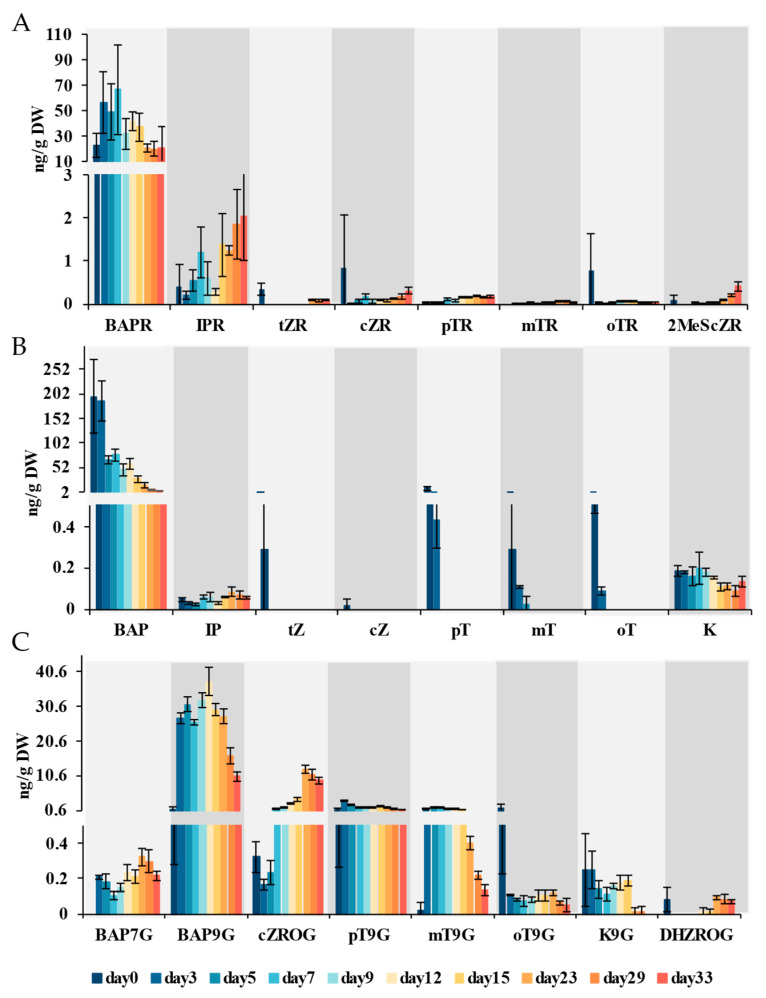
Dynamics of cytokinin precursors (**A**), active form (**B**) and metabolites (**C**) during callus induction and adventitious organogenesis; 6-Benzyladenosine (BAPR), N^6^-isopentenyladenosine (IPR), trans-Zeatin riboside (tZR), cis-Zeatin riboside (cZR), para-Topolin riboside (pTR), meta-Topolin riboside (mTR), ortho-Topolin riboside (oTR), 2-Methylthio-cis-zeatin riboside (2MeScZR), 6-Benzyladenine (BAP), N^6^-isopentenyladenine (IP), trans-Zeatin (tZ), cis-Zeatin (cZ), para-Topolin (pT), meta-Topolin (mT), ortho-Topolin (oT), kinetin (K), N^6^-Benzyladenine-7-glucoside (BAP7G), N^6^-Benzyladenine-9-glucoside (BAP9G), cis-Zeatin-O-glucoside riboside (cZROG), para-Topolin-9-glucoside (pT9G), meta-Topolin-9-glucoside (mT9G), ortho-Topolin-9-glucoside (oT9G), kinetin-9-glucoside (K9G), dihydrozeatin-O-glucoside riboside (DHZROG). Data are shown as mean ± standard deviation (SD) for three independent biological replicates. The concentrations were determined with LC-MS/MS.

Cytokinins function in regulating plant growth and development, as they can promote cell division and the growth of meristematic tissues. In this study, a total of eight precursors of cytokinin (BAPR, IPR, tZR, cZR, pTR, mTR, oTR, 2MeSCZR) were captured (Figure 3A). Among them, high levels of accumulation were detected in 6-benzyladenosine (BAPR) and N6-isopentenyladenosine (IPR), while the remaining six were all below 1 ng/g. BAPR and IPR showed an upward trend during the callus induction stage, reaching their highest levels on the 7th day, at 66.6 ng/g and 1.19 ng/g, respectively. Eight active cytokinins (BAP, IP, tZ, cZ, pT, mT, oT, K) were detected (Figure 3B). Among them, 6-benzylaminopurine (BAP) had a relatively high accumulation level throughout the induction process, showing a sustained decline. However, other active cytokinins were at relatively low levels, all below 1 ng/g, except for the concentration of pT at the beginning of induction. Moreover, eight types of cytokinin glycoside metabolites (BAP7G, BAP9G, cZROG, DHZROG, pT9G, mT9G, oT9G, K9G) were detected (Figure 3C). As a glycoside metabolite of BAP, BAP9G accumulated higher throughout the induction process compared to other cytokinin metabolites. The content of BAP9G suddenly increased from the third day of induction, and reached its highest level of 37.5 ng/g on the 12th day with the appearance of adventitious shoots. Then, the accumulation level of BAP9G continued to decrease.

#### 2.2.3. Gibberellins

**Figure 4:**
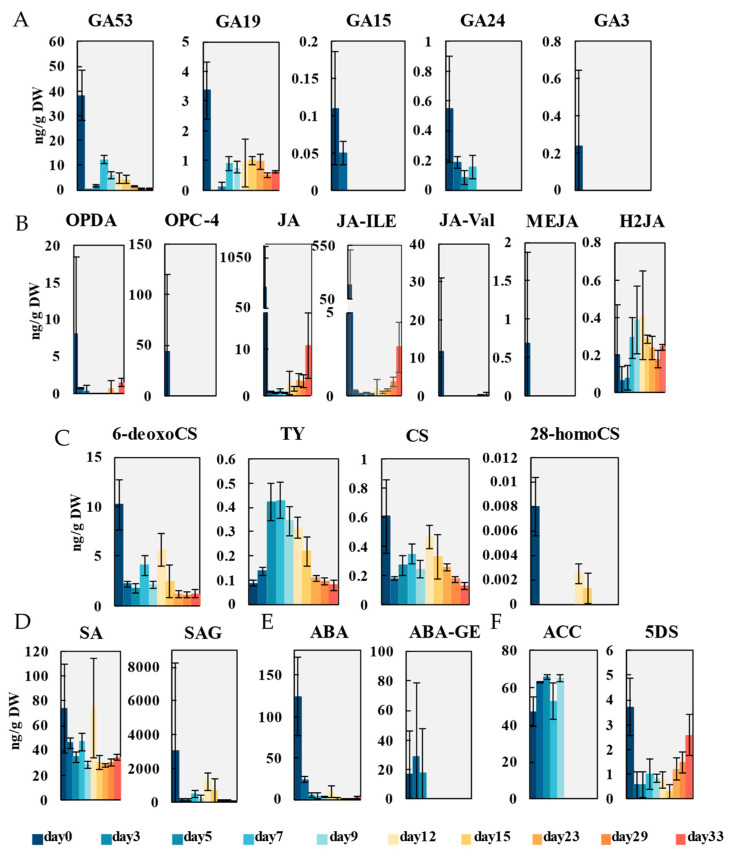
Dynamics of metabolites related to gibberellin (**A**), jasmonic acid (**B**), brassinolide (**C**), salicylic acid (**D**), abscisic acid (**E**) and ethylene (**F**) during callus induction and adventitious organogenesis. Gibberellin A15 (GA15), gibberellin A24 (GA24), gibberellin A53 (GA53), gibberellin A19 (GA19), gibberellin A3 (GA3), 12-oxophytodienoic acid (OPDA), 3-oxo-2-(2-(Z)-Pentenyl) cyclopentane-1-butyric acid (OPC-4), jasmonic acid (JA), jasmonoyl-L-isoleucine (JA-ILE), N-[(-)-Jasmonoyl]-(L)-valine (JA-Val), Methyl jasmonate (MEJA), dihydrojasmonic acid (H2JA), 6-deoxocastasterone (6-deoxoCS), typhasterol (TY), castasterone (CS), 28-homocastasterone (28-hpmpCS), salicylic acid (SA), salicylic acid 2-O-β-glucoside (SAG), abscisic acid (ABA), ABA-glucosyl ester (ABA-GE), 1-Aminocyclopropanecarboxylic acid (ACC), 5-Deoxystrigol (5DS). Data are shown as mean ± standard deviation (SD) for three independent biological replicates. The concentrations were determined with LC-MS/MS.

The active gibberellins (GAs) in plants are GA1, GA3, GA4 and GA7. A total of five gibberellin compounds (GA15, GA24, GA53, GA19, GA3) were identified in this study (Figure 4A). Among them, the contents of GA53 and GA19, precursors of active gibberellin GA1, were higher than others. On the 3rd day of induction, the contents suddenly decreased to 0.02 ng/g and 0 ng/g, respectively, and on day 7, they increased again to 12.3 ng/g and 0.90 ng/g. With the growth of adventitious shoots, the content of GA53 continued to decrease, while the accumulation level of GA19 tended to stabilize. However, the accumulation levels of GA24 and GA15, precursors of active gibberellin GA4, were only detected in trace amounts during the first seven days and three days of induction, respectively. Active gibberellin GA3 was only detected at a concentration of 0.24 ng/g at the beginning of induction, and was not detected subsequently.

#### 2.2.4. Jasmonic Acid

Jasmonic acid (JA), as a signaling molecule, has regulatory effects on plant growth, development and environmental stress. The main synthetic pathways are the octadecane pathway starting from alpha linolenic acid (18:3) and the hexadecane pathway starting from hexadecanoic acid (16:3). Additionally, there may be a pathway from linoleic acid to dihydrojasmonic acid in plants [32]. Analysis of JA during callus induction and adventitious organogenesis of *P. alba* showed that a total of seven JA-related compounds (OPDA, OPC-4, JA, JA-ILE, JA Val, MEJA, H2JA) were detected (Figure 4B). Among them, OPDA and OPC-4, involved in the octadecane pathway, had high accumulation after wounding, at 8.12 ng/g and 44.1 ng/g, respectively. The content of JA was initially found to be 470 ng/g, followed by a sudden decrease. Along with the growth of callus and adventitious shoots, JA steadily increased, and on day 33, the content was 10.9 ng/g. JA-ILE, JA-Val and MEJA are metabolites of JA, which were accumulated within a short period of time after wounding. The content of JA-ILE was much higher than that of JA-Val and MEJA. On the third day of induction, the JA-ILE content rapidly decreased to 0.27 ng/g. With the growth of callus and adventitious shoots, the JA-ILE level gradually accumulated to a content of 2.92 ng/g. In addition, although dihydrojasmonic acid (H2JA) was detected during the induction process, its overall content was relatively low. However, compounds associated with the hexadecanoic acid (16:3) pathway were not detected. In the early stage of induction, the JA signaling pathway may be activated through the octadecane pathway.

#### 2.2.5. Brassinolide

Brassinolide (BR) not only regulates the growth of lateral buds by affecting the synthesis and distribution of IAA, but also inhibits the lateral bud dormancy regulatory gene BRANCHED1 (BRC1) through the BR signaling pathway, relieving the inhibitory effect of BRC1 on lateral bud development. In this study, four BR compounds (6-deoxoCS, TY, CS, 28-homoCS) were extracted (Figure 4C). The biosynthetic pathways of BR can be divided into early C-6 oxidation pathway and late C-6 oxidation pathway. The 6-deoxocastasterone (6-deoxoCS) is a precursor of castasterone (CS) and participates in the late C-6 oxidation pathway. Its accumulation level during callus and adventitious shoot induction was higher than the other three brassinosteroids. Its content fluctuated with the process of organogenesis, such as two increases after two decreases on the 7th and 12th days for callus and adventitious shoot induction, respectively. Meanwhile, typhasterol (TY) can also be oxidized to CS and participate in the early C-6 oxidation pathway. The accumulation level of TY was relatively low throughout the induction process. TY gradually accumulates during the callus induction, with the highest content reaching 0.42 ng/g on the 7th day. During the adventitious shoot induction, the accumulation level of TY gradually decreases. CS, as a necessary precursor in various pathways of BR synthesis, was initially found to have its highest content of 0.61 ng/g, and rapidly decreased to 0.18 ng/g in the following 3 days. With the appearance of callus, the accumulation level of CS gradually increased. On day 12, with the emergence of adventitious shoots, the CS content reached 0.46 ng/g. Continuing to induce the growth of adventitious shoots, the CS content gradually decreased to the level of day 3. The 28-high brassinoketone (28-homoCS) with BR function was detected only in trace amounts on days 0, 12 and 15.

#### 2.2.6. Other Hormones

In addition to the hormones mentioned above, salicylic acid (SA), abscisic acid (ABA), ethylene and strigolactones (SLs) are also important regulators of plant growth and development. The accumulation of two salicylic acid compounds (SA, SAG) were found under callus induction and adventitious organogenesis of *P. alba* (Figure 4D). The levels of SA and its glycoside derivative SAG initially accumulated significantly, reaching 74.2 ng/g and 3080 ng/g, respectively. They both showed two increases, corresponding to the timing of callus and adventitious shoot emergence, respectively. Then, their content decreased again and eventually stabilized.

The content of active abscisic acid (ABA) also showed a peak at the beginning of induction, and then rapidly decreased (Figure 4E). Along with the appearance of adventitious shoots, the ABA content slightly increased, reaching 9.35 ng/g on day 12, and then decreased again with the growth of adventitious shoots. ABA glycosyl ester (ABA-GE), as an inactive form of ABA, was only detected within 5 days before adventitious shoots appeared, and the highest content was 28.9 ng/g on the third day.

In addition, ethylene also has the function of promoting flower bud differentiation. One ethylene compound (ACC) was also detected during the induction of callus and adventitious shoots (Figure 4F). ACC is a precursor substance of ethylene, which accumulated only within 9 days of callus induction. On the third day, the highest ACC content was 63 ng/g. After further cultivation for 7 days, ACC decreased again to 52.8 ng/g, and then increased again to 65.2 ng/g on the 9th day. It was not detected during the subsequent adventitious shoot induction process.

SLs are divided into typical SL and atypical SL. Typical SL contains a tricyclic lactone (ABC ring) and a monocyclic lactone (D ring) connected by an enol ether bond, while atypical SL has an unclosed BC ring. A typical SL (5DS) was detected (Figure 4F). The highest content of 5DS was 3.72 ng/g immediately after wounding. Subsequently, the accumulation level of 5DS remained low during callus and adventitious shoot induction, reaching a minimum of 0.32 ng/g on day 15. With the growth of adventitious shoots, the accumulation level of 5DS slowly increased, reaching 2.58 ng/g on day 33.

### 2.3. Changes in Metabolic Enzyme Activity During the Regeneration Process of P. alba

The levels of endogenous auxin and cytokinin are closely related to the induction of callus and organ differentiation. To further clarify the process of callus and adventitious shoot formation in the stem segments of *P. alba*, the activity of three endogenous proteases closely related to auxin and cytokinin metabolism were evaluated.

**Figure 5:**
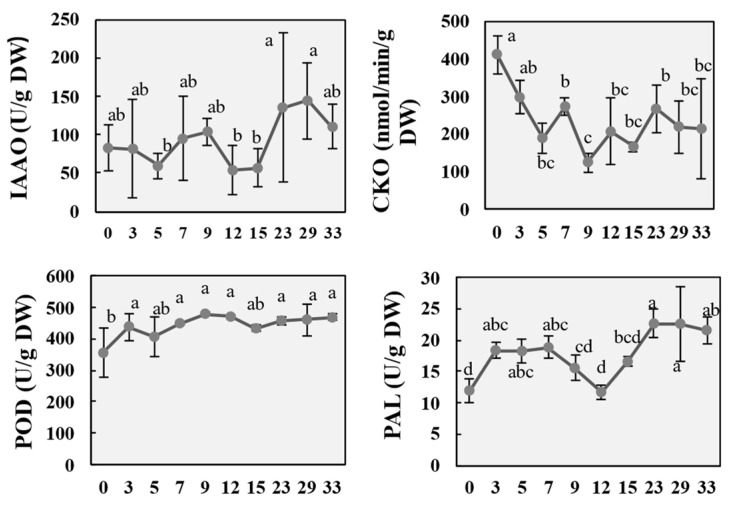
Activities of IAAO (indole-3-acetic acid oxidase), CKO (cytokinin oxidase/dehydrogenase), POD (peroxidase) and PAL (phenylalanine oxidase) during callus induction and adventitious organogenesis. Data are shown as mean ± standard deviation (SD) for five independent biological replicates. The letters represent the statistical significance determined by analysis of variance in Tukey’s multiple comparison test *(p* ≤ 0.01).

Indole-3-acetic acid oxidase (IAAO) is a key enzyme in IAA catabolism, capable of oxidizing and decomposing IAA to inactivate it [33]. The IAAO activity analysis (Figure 5) showed that during the initial stage of callus induction, the IAAO activity gradually decreased, reaching a minimum of 59.3 U/g on the 5th day. With the formation of callus, IAAO activity increased from day 7 and reached a concentration of 104 U/g on day 9. The IAAO activity suddenly decreased to 53.7 U/g on the 12th day. With the proliferation of callus tissue and the growth of adventitious shoots, IAAO activity rapidly increased on the 23rd day and reached its highest level of 144 U/g on the 29th day.

Cytokinin has a synergistic effect with auxin, and cytokinin oxidase/dehydrogenase (CKO) is the rate-limiting enzyme in the cytokinin degradation pathway [34]. CKO activity analysis showed that CKO had the highest activity at 411 nmol/min/g immediately after wounding (Figure 5). The CKO activity rapidly decreased during the callus induction. With the appearance of callus tissue, CKO activity transitory increased and then decreased to a minimum of 125 nmol/min/g on the 9th day. When adventitious shoots appeared, CKO activity increased again, reaching 206 nmol/min/g on the 12th day. Along with the development of adventitious shoots, CKO activity increased to 267 nmol/min/g on the 23rd day. From day 23 to day 33, CKO activity slightly decreased and gradually stabilized. During the entire process of callus induction and adventitious shoot regeneration, CKO activity showed a decreasing trend and fluctuated with the organogenesis.

Peroxidase (POD), as an antioxidant enzyme in plants, not only regulates reactive oxygen species metabolism but also participates in cell division and differentiation. It catalyzes the oxidation and decarboxylation of IAA [35]. The analysis of peroxidase (POD) activity showed that during the induction of callus, POD activity gradually increased and reached its highest level of 480 U/g on the 9th day (Figure 5). With the emergence of adventitious shoots, POD activity showed a slow downward trend, and then increased again after 15 days.

In addition, phenylalanine oxidase (PAL) is a key enzyme in the synthesis pathway of various important secondary metabolites such as lignin, flavonoids, alkaloids, etc. During differentiation, PAL activity usually increases with cell division [36]. In this study, PAL activity rapidly increased three days after callus induction and then stabilized at around 18.9 U/g (Figure 5). After 7 days of induction, PAL activity decreased with the proliferation of callus tissue and the appearance of adventitious shoots. After 12 days of stem segment inoculation, PAL activity suddenly increased to 22.6 U/g and then stabilized.

### 2.4. Transcriptional Regulation Callus Induction and Adventitious Organogenesis of P. alba

**Figure 6:**
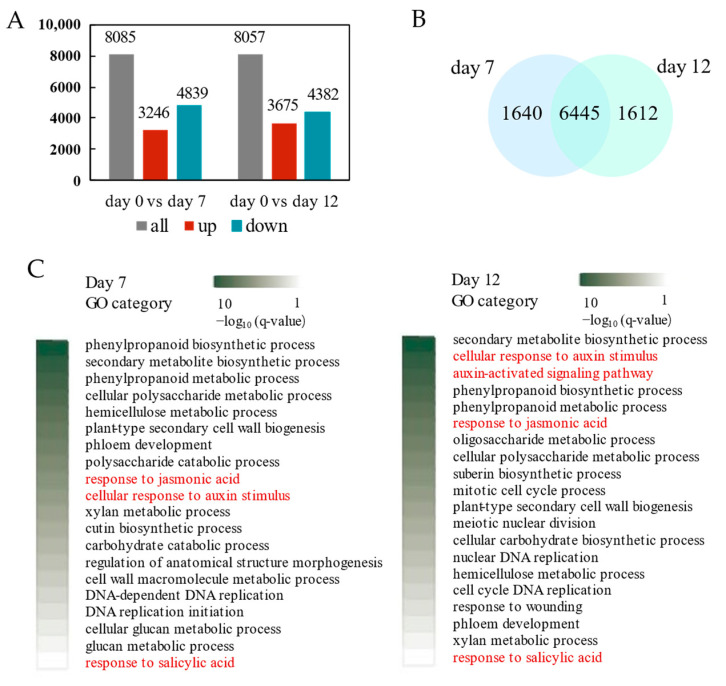
Regulation of gene expression during callus induction and adventitious organogenesis. (**A**) Differentially expressed genes (DEGs) in *P. alba* induced for 7 and 12 days. (**B**) Venn diagram of DEGs induced for 7 and 12 days. (**C**) The top twenty most significantly enriched GO terms. Hormone response terms are colored in red.

After seven days of inoculation, callus appeared on the stem of *P. alba*, and after twelve days of inoculation, adventitious shoots appeared. Endogenous hormone analysis also showed significant changes in these two stages. To explore the transcriptome changes during the callus tissue and adventitious shoot regeneration process of *P. alba*, transcriptomes at 7 and 12 days after induction were compared with the transcriptome at day 0, respectively. In total, 8085 and 8057 differentially expressed genes (DEGs) were identified after 7 days (day 7 vs. day 0) and 12 days (day 12 vs. day 0) of inoculation (Figure 6A). Among them, 1640 specific genes contributed to callus formation, while 1612 genes might have played a role in the induction of adventitious shoots (Figure 6B). GO enrichment analysis of DEGs showed that the two developmental processes were both influenced by hormone-related pathway (Figure 6C), such as cellular response to auxin stimulus, auxin-activated signaling pathway, response to jasmonic acid, and response to salicylic acid. In addition, GO terms involved in the biosynthesis process of cellular polysaccharide, hemicellulose, phenylpropanoid, xylan and phloem, which related to cell wall synthesis, were significantly enriched.

### 2.5. Corresponding Hormone Synthesis and Metabolism Pathways

**Figure 7:**
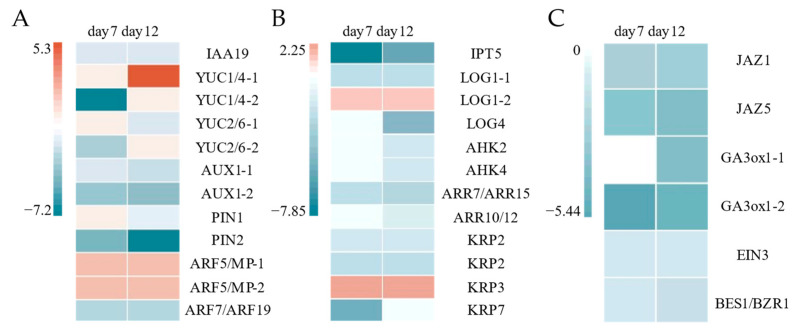
Heat map of gene expression profiles for metabolism of auxin (**A**), cytokinin (**B**), jasmonic acid, gibberellin, ethylene and brassinosteroid (**C**) during callus induction and adventitious organogenesis of *P. alba*.

Based on the transcriptome analysis and previous research, expression patterns of regulation genes involved in plant hormone synthesis and signal transduction were identified (Figure 7). Among them, *YUC1*/*4*-*1* (*Poalb06G007060*), *ARF5*/*MP*-*1* (*Poalb05G019510*) and *ARF5*/*MP*-*2* (*Poalb02G002250*) were up-regulated during the callus induction, while the expression of other genes was down-regulated or remained unchanged (Figure 7A). The auxin-related genes were not only limited to GO terms such as auxin-activated signaling pathway and cellular response to auxin stimulus, but also significantly enriched in developmental GO terms such as plant epidermis development, lateral root development, shoot system morphogenesis and plant organ formation (Table S1). These results indicate that the tryptophan (Trp) auxin synthesis pathway may be activated during the callus induction process of *P. alba*, and plays an important role in callus and adventitious shoot induction.

Among cytokinin metabolism-related genes, *LONELY GUY 1* (*LOG1*)(*Poalb04G017770*) and *KIP*-*RELATED PROTEIN 3* (*KRP3*)(*Poalb02G015980*) were up-regulated for adventitious organogenesis, while the expression of other genes was down-regulated or remained unchanged (Figure 7B). Cytokinin plays an important regulatory role in cell proliferation, differentiation and development [37]. The cytokinin-related genes were mainly enriched in hormone metabolic processes and hormone biosynthetic processes, as well as GO terms related to the cell cycle, such as regulation of the cell cycle, DNA replication and cell cycle DNA replication (Table S2). It suggests that cytokinins induce the formation of callus by affecting cell proliferation.

Other hormone-related genes have also been identified, such as jasmonic acid receptors *JASMONATE ZIM-DOMAIN PROTEIN 1* (*JAZ1*) (*Poalb06G022000*), *JAZ5* (*Poalb03G005440*), gibberellin synthase gene *GA3*-*oxidase1* (*GA3ox1*) (*Poalb01G015870*, *Poalb03G004570*), ethylene-signaling gene *ETHYLENENSENSITIVE3* (*EIN3*) (*Poalb04G016640*), and brassinosteroid signaling pathway gene *BRI1EMS SUPERSOR 1* (*BES1*)/*BRASSINAZOLE RESISTANT 1* (*BZR1*) (*Poalb02G007780*). The expression of these genes was down-regulated for callus induction (Figure 7C).

The results show that auxin and cytokinin are not only the key hormones in callus induction, but also reveal the response of auxin and cytokinin metabolism to organ regeneration in *P. alba*. Fifteen DEGs involved in auxin and cytokinin mediated regulation for regeneration were identified in *P. alba* (Table 1).

## 3. Discussion

### 3.1. Synergistic Effects of Multiple Hormones During Callus Induction in Stem of Populus alba

In *P. alba*, callus could be induced from the stem within 7 days, and adventitious shoots occurred on the 12th day of induction. Auxins and cytokinins are closely related to callus and adventitious shoot formation [38]. Low concentrations of IAA can promote cell division, while high concentrations of IAA have inhibitory effects [39]. Previous research showed that auxin response signals initially exist in small cell clusters that proliferate to form callus tissue, but weaken in callus, indicating that auxin response is only necessary in early cell proliferation during callus induction [40]. In the first week of induction, cells underwent dedifferentiation and extensive division. Disregarding the transient hormone burst induced by wounding (Figure S1), the precursors Trp and ICAld of auxin and the storage forms of IAA (IAA-Glc, IAA-Ala and IAA-Gly) increased during the callus induction. The Trp-dependent IAA synthesis pathway is likely activated, supported by evidence of the depletion of IAA storage forms (Figure 2C) and the up-regulation of auxin biosynthesis gene *YUC1/4-1* and the auxin signaling factor *ARF5* (Figure 7A). The IAA oxidase content was correspondingly reduced. This indicates that the Trp-dependent auxin synthesis pathway was rapidly activated in the early stage of callus induction, and the IAA metabolic pathway has been inhibited.

Cytokinins also play an important role in the proliferation of callus cells [41]. In this study, the accumulation of cytokinins was simultaneous with callus formation. The cytokinin BAP with highest content and various active endogenous cytokinins pT, mT and oT were all down-regulated. IP and K do not seem to be involved in regulating callus formation, as their levels are relatively stable during induction. Extensive studies have shown that a high ratio of cytokinin to auxin is not conducive to induce callus formation, while a high-cytokinin-to-low-auxin ratio is required for adventitious shoot regeneration [42]. In this study, we employed a medium with a relative high concentration of cytokinin (0.2 mg/L 6-BA). The results showed that during the callus induction phase (days 0–7), the concentration of active cytokinin (BAP) progressively declined, accompanied by the accumulation of its corresponding precursor (BAPR) and inactive metabolites (BAP7G and BAP9G). This metabolic shift indicates tight regulation of cytokinin homeostasis, with pathway flux redirected toward upstream and downstream branches. During adventitious shoot regeneration (after day 12), despite sustained exogenous 6-BA supply, precursor, active and metabolite pools were depleted, suggesting massive cytokinin demand for organogenesis. However, more detailed and conclusive evidence is necessary for verification.

JA and ABA are key endogenous mediators during abiotic stress and defense responses [43]. JA and ABA are defined to inhibit the formation of callus tissue, and the content of JA increases after wounding and then decreases [44]. The results in this study revealed similar sharp decreases in the contents of active JA and ABA. Although the content of brassinosteroid precursors (6-deoxoC and CS) was relatively low, it rapidly decreased after wounding and then showed an upward trend during callus formation (day 3 to day 12). BR has a positive regulatory effect on cell division [45], and can also co-regulate callus formation with auxin. These results suggest that auxin, cytokinin, JA, ABA, GA and BR all have regulatory effects in the callus induction process of *P. alba*, while functions of SA, ethylene and SLs are not significant.

### 3.2. Synergistic Induction of Adventitious Shoots by Multiple Hormones in Stem of P. alba

Auxin is believed to play a critical role in the transformation of cell fate, enabling callus to form adventitious shoots [46,47]. In *P. alba*, the accumulation of auxin precursor Trp and active auxin ICAld showed an upward trend during adventitious shoot formation. The storage forms of auxin, IAA-Ala and IAA-Gly, as well as the irreversible metabolites IAA-Glu and IAA-Asp, gradually increased. At the same time, the content of IAAO did not show significant fluctuations during the induction of adventitious shoots, indicating that the Trp-dependent auxin synthesis pathway and the metabolic pathways through IAA-Ala, IAA-Gly, IAA-Glu and IAA-Asp were activated. Cytokinin is another important factor in regulating plant growth and development, and a high ratio of cytokinin/auxin induces shoot formation [48]. Cytokinin metabolites BAP7G/9G accumulated significantly during the induction of adventitious shoots, with the highest content of BAP7G at day 23 and BAP9G at day 12, indicating that the BAP metabolic pathway was activated. Other plant hormones that affect the process of adventitious shoot regeneration include BR and GA [49]. BR mainly regulates cell division and differentiation, and BR precursors (6-deoxoC and CS) have a high accumulation in the initial stage of adventitious shoot induction, further confirming the role of BR in regulating adventitious shoot regeneration. On the contrary, GA inhibits the regeneration of adventitious shoots [50]. No accumulation of active GA was found during the induction of adventitious shoots, which further confirmed the divergent roles of various hormones for adventitious organogenesis in *P. alba*.

### 3.3. Auxin- and Cytokinin-Mediated Regulation in Callus Induction and Adventitious Organogenesis of P. alba

**Figure 8:**
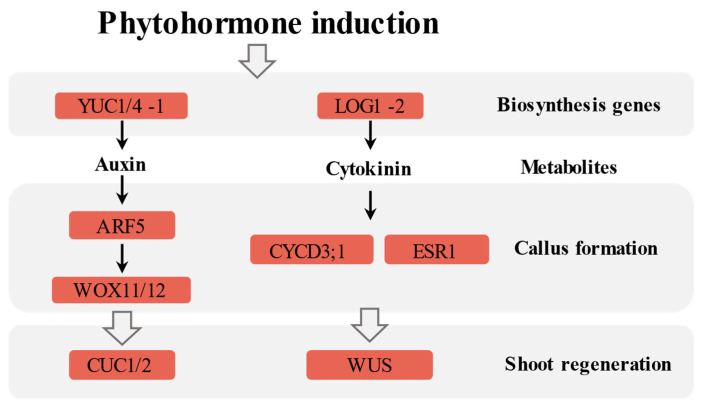
Auxin- and cytokinin-mediated regulation in callus induction and adventitious organogenesis process of *P. alba*.

During the induction of callus and adventitious shoots in *P. alba*, auxin and cytokinin play a major regulatory role. Based on the results, an induction process regulated by auxin and cytokinin is proposed in this study (Figure 8).

YUCCA (YUC)-mediated auxin biosynthesis genes are essential for organ formation [51]. The expression of *YUC1*/*4*-*1* (*Poalb06G007060*) in *P. alba* was up-regulated on the 12th day of induction, confirming the initiation of auxin synthesis and helping to maintain the content of auxin before meristem initiation. The formation of callus was induced by auxin, which is similar to the process of lateral root initiation, and exhibits root meristematic characteristics [52,53]. Auxin activates the expression of *WUSCHEL-RELATED HOMOBOX11*/*12* (*WOX11*/*12*) transcription factor genes through signal transduction, marking the transition of regenerative potential cells to root founder cells [54,55]. *WOX11*/*12* and the auxin signaling factor *ARF5* were up-regulated on the 7th and 12th days of callus induction, indicating the activation of the auxin signaling pathway at this stage, and the fate of cells with regenerative potential began to change. CUPSHAPED COTYLEDON1/2 (CUC1/2) is essential for the initiation of SAM [56,57]. *CUC1*/*2* showed no significant difference in expression after 7 days of callus induction, but was significantly up-regulated after 12 days of development. This indicated the onset of adventitious shoot primordia after 12 days of induction.

The *LOG1*, *LOG4* and *LOG5* genes encode enzymes involved in the biosynthesis of cytokinins, which are activated during plant regeneration [58]. The expression of *P. alba LOG1*-*2* (*Poalb04G017770*) was up-regulated on the 7th and 12th day of induction, indicating that the synthesis of cytokinins might be activated by *LOG1*-*2*. ENHANCER OF SHOOT REGENERATION 1 (ESR1) is a key regulatory factor in the formation of callus and adventitious shoots [37,59,60,61]. WUSCHEL (WUS) is an important regulatory factor for de novo shoot regeneration [62]. It specifically controls the *WUS*/*CLV3* feedback loop through repressing the expression of *ARR7* and *ARR15* in SAM, which is crucial for maintaining SAM stem cell activity [63]. Both *ESR1* and *WUS* were up-regulated, indicating that the regeneration of adventitious buds was initiated during the formation of callus in *P. alba*. *Cyclin D3*;*1* (*CYCD3*;*1*) was up-regulated, while *CYCD1*;*1* was down-regulated. The up-regulated expression of *CYCD3*;*1* indicates the re-entry of the cell cycle.

These results imply that auxin may promote the formation of callus in *P. alba* by activating the expression of *WOX11*/*12*. By up-regulating the expression of *CUC1*/*2*, the development of callus begins to initiate SAM at day 12. The cytokinin-mediated pathway regulates the adventitious shoot formation by ESR1 and WUS. The key regulators identified in this study will accelerate the exploration and understanding of the asexual reproduction mechanism of poplar trees.

## 4. Materials and Methods

### 4.1. Plant Material and Induction of Callus and Shoot Regeneration

The genome-sequenced *Populus alba* genotype [64] was propagated by tissue culture. The *P. alba* seedlings were initially cultured on 1/2 Murashige and Skoog (MS) solid medium (0.1 mg/L IBA, 15.0 g/L sucrose, 6.0 g/L agar, pH 5.8–6.0) [65], under controlled conditions of 22 ± 2 °C, 4500 lux light intensity and 16 h light/8 h dark photoperiod for propagation. After 4 weeks of growth, internodal stem segments from uniformly grown seedlings were sectioned into 3–4 mm explants under sterile conditions. These stem cuttings were then transferred to MS medium (0.1 mg/L NAA, 0.2 mg/L 6-BA, 30.0 g/L sucrose, 6.0 g/L agar at pH 5.8–6.0) for callus induction and adventitious shoot regeneration [65]. The stem cuttings were collected on 0, 3, 5, 7, 9, 12, 15, 23, 29 and 33 days for endogenous hormone quantification and physiological assays. Three biological replicates per time point were analyzed for hormonal profiles, while five replicates were allocated to physiological index measurements.

### 4.2. Quantitative Analysis of Plant Hormone

Stem explant hormone level analysis was performed by Metware Biotech (Wuhan, China), with three biological replicates per time point and 300 mg per replicate. Fresh plant sample was harvested, immediately frozen in liquid nitrogen, ground into powder, dissolved in 1 mL methanol/water/formic acid (15:4:1, *v*/*v*/*v*). A total of 10 μL internal standard mixed solution (100 ng/mL) was added into the extract as internal standards for the quantification. The mixture was vortexed for 10 min, then centrifuged for 5 min, 12,000 rpm; the supernatant was transferred to clean plastic microtubes, followed by evaporation to dryness and dissolved in 100 μL 80% methanol (*v*/*v*), and filtered through a 0.22 μm membrane filter for further LC-MS/MS analysis. The sample extracts were analyzed using a UPLC-ESI-MS/MS system, AB Sciex, Framingham, MA, USA (UPLC, ExionLC™ AD; MS, 6500 Triple Quadrupole). The ESI source operation parameters were as follows: ion source, ESI+/−; source temperature 550 °C; ion spray voltage (IS) 5500 V, −4500 V (Negative); curtain gas (CUR) was set at 35 psi, respectively. Phytohormones were analyzed using scheduled multiple reaction monitoring (MRM).

### 4.3. Metabolic Enzyme Activity

Peroxidase (POD) was extracted using a peroxidase (POD) assay kit (Nanjing Jiancheng, Nanjing, China). Phenylalanine oxidase (PAL) was extracted using a phenylalanine oxidase (PAL) assay kit (Nanjing Jiancheng, Nanjing, China). Cytokinin oxidase/dehydrogenase (CKO) was extracted using a cytokinin oxidase/dehydrogenase (CKO) assay kit (Geruisi, Suzhou, China). Indole-3-acetic acid oxidase (IAAO) was extracted using an Indole-3-acetic acid oxidase (IAAO) assay kit (Boxbio, Beijing, China). There were five biological replicates per time point and 0.1 g of stem segment explants per replicate.

### 4.4. RNA-Seq Analysis

For RNA-seq, samples from different developmental stages (day 0, day 7 and day 12) of stem explants were collected. Three biological replicates were used in this study. RNA isolation, library construction and sequencing were carried out at Biomarker Technologies (Beijing, China). Raw reads were filtered to obtain clean reads using Fastp (V0.20.1) software. The clean reads were aligned with the *P. alba* genome (Palba_v2.0) obtained using the HISAT (V2.2.1) software. The readcounts were quantified using StringTie (V2.1.3b). The differentially expressed genes (DEGs) were identified using the DESeq2 package (V1.26.0). Genes with an absolute fold change (FC) ≥ 2 and a padj < 0.05 were considered differentially expressed. GO enrichment analysis of the DEGs was conducted using the ClusterProfiler (V3.14.3).

### 4.5. Identification of Hormones and Development-Related Genes

Gene families related to hormone metabolism and plant development in *Arabidopsis thaliana* (Athaliana_TAIR10) and *Populus trichocarpa* (Ptrichocarpa_v4.1) genomes were downloaded from Phytozome V13 (https://phytozome.jgi.doe.gov, accessed on 21 December 2021). These genes were used as templates to search for candidate hormones and development-related genes in *P. alba* using BLAST (V2.13.0).

## 5. Conclusions

In this study, by combining the results of endogenous hormone contents, physiological characteristics and transcriptional profiles, we proposed a regeneration pathway regulated by auxin and cytokinin for poplars. *YUC1*/*4-1*, *WOX11*/*12* and *ARF5* promote the formation of callus by activating the auxin signaling pathway, while also initiating the fate transition of cells with regeneration potential. By up-regulating the expression of *CUC1/2*, the development of callus begins to initiate SAM. The cytokinin-mediated pathway regulates the de novo shoot formation by *LOG1-2*, *ESR1*, *WUS* and *CYCD3;1*. The precursors of active gibberellin GA1, GA53 and GA19 were accumulated in the early stage of callus induction, and then they continued to decrease. JA may function on adventitious shoot regeneration due to its accumulation after 12 days of induction. Future research and functional verification are needed to reveal the molecular regulatory mechanism of each hormone component in callus induction and adventitious shoot regeneration of poplars.

## Figures and Tables

**Table 1:** DEGs involved in auxin and cytokinin-mediated regulation of callus induction and adventitious organogenesis of *P. alba*.

Gene ID	Description	Log_2_FC	Log_2_FC	Process
		(0 vs. 7)	(0 vs. 12)	
*Poalb02G008940*	*LBD18*; *LOB DOMAIN-CONTAINING PROTEIN 18*	−3.3	0	Auxin signaling
*Poalb14G005550*	*LBD18*; *LOB DOMAIN-CONTAINING PROTEIN 18*	−4.59	−2.09	Auxin signaling
*Poalb01G034320*	*CUC1/2*; *CUP*-*SHAPED COTYLEDON1*/*2*	0	6.71	Auxin/Cytokinin signaling
*Poalb11G007910*	*CUC1/2*; *CUP*-*SHAPED COTYLEDON1*/*2*	0	10.18	Auxin/Cytokinin signaling
*Poalb13G011170*	*WOX11/12*; *WUSCHEL*-*RELATED HOMEOBOX 11*/*12*	9.87	10.14	Auxin signaling
*Poalb06G000150*	*PLT5*; *AP2 family of transcriptional regulators*	0	−1.2	Auxin signaling
*Poalb18G002670*	*PLT5*; *AP2 family of transcriptional regulators*	0	−1.22	Auxin signaling
*Poalb07G000880*	*bZIP59*	0	−1.01	Auxin signaling
*Poalb05G009480*	*WUSCHEL*	2.29	3.09	Cytokinin signaling
*Poalb08G016150*	*ESR1*; *ENHANCER OF SHOOT REGENERATION 1*	0	4.42	Cytokinin signaling
*Poalb10G003790*	*ESR1*; *ENHANCER OF SHOOT REGENERATION 1*	10.39	10.36	Cytokinin signaling
*Poalb08G012860*	*CYCD1;1*	−2.01	−2.06	Cytokinin signaling
*Poalb10G008040*	*CYCD1;1*	−1.81	−1.7	Cytokinin signaling
*Poalb01G026650*	*CYCD3;1*	3.13	2.48	Cytokinin signaling
*Poalb09G008730*	*CYCD3;1*	3.91	3.17	Cytokinin signaling

## Data Availability

RNA-seq data generated in this study have been deposited in National Genomics Data Center, Beijing Institute of Genomics, Chinese Academy of Sciences / China National Center for Bioinformation, under the BioProject PRJCA035821.

## Supplementary Materials

The following supporting information can be downloaded at: https://www.mdpi.com/article/10.3390/ijms26094087/s1.

## Abbreviations

The following abbreviations are used in this manuscript:

TRP	L-tryptophan
IAA	Indole-3-acetic acid
IAA-Ala	IAA-alanine
IAA-Asp	IAA-L-aspartate
IAA-Glc	IAA-glucose
IAA-Glu	IAA-L-glutamate
IAA-Gly	IAA-glycine
IBA	Indole-3-butyric acid
ICAld	Indole-3-carboxaldehyde
MEIAA	Methylindole-3-acetic acid
OxIAA	2-oxindole-3-acetic acid
BAPR	6-Benzyladenosine
IPR	N6-isopentenyladenosine
2MeScZR	2-Methylthio-cis-zeatin riboside
BAP	6-Benzyladenine
BAP7G	N6-Benzyladenine-7-glucoside
BAP9G	N6-Benzyladenine-9-glucoside
cZ	cis-Zeatin
cZR	cis-Zeatin riboside
cZROG	cis-Zeatin-O-glucoside riboside
DHZROG	Dihydrozeatin-O-glucoside riboside
IP	N6-isopentenyladenine
K	Kinetin
K9G	Kinetin-9-glucoside
mT	Meta-Topolin
mT9G	meta-Topolin-9-glucoside
mTR	meta-Topolin riboside
oT	ortho-Topolin
oT9G	ortho-Topolin-9-glucoside
oTR	ortho-Topolin riboside
pT	para-Topolin
pT9G	para-Topolin-9-glucoside
pTR	para-Topolin riboside
tZ	trans-Zeatin
GA3	Gibberellin A3
GA15	Gibberellin A15
GA19	Gibberellin A19
GA24	Gibberellin A24
GA53	Gibberellin A53
H2JA	Dihydrojasmonic acid
JA	Jasmonic acid
JA-ILE	Jasmonoyl-L-isoleucine
JA-Val	N-[(-)-Jasmonoyl]-(L)-valine
MEJA	Methyl jasmonate
OPC-4	3-oxo-2-(2-(Z)-Pentenyl) cyclopentane-1-butyric acid
OPDA	12-oxophytodienoic acid
28-hpmpCS	28-homocastasterone
6-deoxoCS	6-deoxocastasterone
CS	Castasterone
TY	Typhasterol
SA	Salicylic acid
SAG	Salicylic acid 2-O-β-glucoside
ABA	Abscisic acid
ABA-GE	ABA-glucosyl ester
ACC	1-Aminocyclopropanecarboxylic acid
5DS	5-Deoxystrigol
CKO	Cytokinin oxidase/dehydrogenase
IAAO	Indole-3-acetic acid oxidase
PAL	Phenylalanine oxidase
POD	Peroxidase

## References

[1] Du X.M., Fang T., Liu Y., Huang L.Y., Zang M.S., Wang G.Y., Liu Y.J., Fu J.J. (2019). Transcriptome profiling predicts new genes to promote maize callus formation and transformation. Front. Plant Sci..

[2] Sugimoto K., Gordon S.P., Meyerowitz E.M. (2011). Regeneration in plants and animals: Dedifferentiation, transdifferentiation, or just differentiation?. Trends Cell Biol..

[3] Sugiyama M. (2015). Historical review of research on plant cell dedifferentiation. J. Plant Res..

[4] Xu L., Huang H. (2014). Genetic and epigenetic controls of plant regeneration. Curr. Top. Dev. Biol..

[5] Chandler J.W. (2011). Founder cell specification. Trends Plant Sci..

[6] Atta R., Laurens L., Boucheron-Dubuisson E., Guivarc’h A., Carnero E., Giraudat-Pautot V., Rech P., Chriqui D. (2009). Pluripotency of *Arabidopsis* xylem pericycle underlies shoot regeneration from root and hypocotyl explants grown in vitro. Plant J..

[7] Yang W., Choi M.H., Noh B., Noh Y.S. (2020). De novo shoot regeneration controlled by HEN1 and TCP3/4 in *Arabidopsis*. Plant Cell Physiol..

[8] Zhang T.Q., Lian H., Zhou C.M., Xu L., Jiao Y.L., Wang J.W. (2017). A two-step model for de novo activation of *WUSCHEL* during plant shoot regeneration. Plant Cell.

[9] Duclercq J., Sangwan-Norreel B., Catterou M., Sangwan R.S. (2011). De novo shoot organogenesis: From art to science. Trends Plant Sci..

[10] Liu J., Hu X.M., Qin P., Prasad K., Hu Y.X., Xu L. (2018). The *WOX11-LBD16* pathway promotes pluripotency acquisition in callus cells during de novo shoot regeneration in tissue culture. Plant Cell Physiol..

[11] Fan M.Z., Xu C.Y., Xu K., Hu Y.X. (2012). LATERAL ORGAN BOUNDARIES DOMAIN transcription factors direct callus formation in *Arabidopsis* regeneration. Cell Res..

[12] Xu C., Luo F., Hochholdinger F. (2016). LOB domain proteins: Beyond lateral organ boundaries. Trends Plant Sci..

[13] Xu C., Cao H., Zhang Q., Wang H., Xin W., Xu E., Zhang S., Yu R., Yu D., Hu Y. (2018). Control of auxin-induced callus formation by bZIP59-LBD complex in *Arabidopsis* regeneration. Nat. Plants.

[14] Huang K.L., Ma G.J., Zhang M.L., Xiong H., Wu H., Zhao C.Z., Liu C.S., Jia H.X., Chen L., Kjorven J.O. (2018). The ARF7 and ARF19 transcription factors positively regulate *PHOSPHATE STARVATION RESPONSE1* in *Arabidopsis* Roots. Plant Physiol..

[15] Pandey S.K., Lee H.W., Kim M.J., Cho C., Oh E., Kim J. (2018). LBD18 uses a dual mode of a positive feedback loop to regulate ARF expression and transcriptional activity in *Arabidopsis*. Plant J..

[16] Zhang S., Yu R., Yu D., Chang P., Guo S., Yang X., Liu X., Xu C., Hu Y. (2022). The calcium signaling module CaM-IQM destabilizes IAA-ARF interaction to regulate callus and lateral root formation. Proc. Natl. Acad. Sci. USA.

[17] Hofhuis H., Laskowski M., Du Y.J., Prasad K., Grigg S., Pinon V., Scheres B. (2013). Phyllotaxis and rhizotaxis in *Arabidopsis* are modified by three *PLETHORA* transcription factors. Curr. Biol..

[18] Pinon V., Prasad K., Grigg S.P., Sanchez-Perez G.F., Scheres B. (2013). Local auxin biosynthesis regulation by PLETHORA transcription factors controls phyllotaxis in *Arabidopsis*. Proc. Natl. Acad. Sci. USA.

[19] Ikeuchi M., Ogawa Y., Iwase A., Sugimoto K. (2016). Plant regeneration: Cellular origins and molecular mechanisms. Development.

[20] Iwase A., Mitsuda N., Koyama T., Hiratsu K., Kojima M., Arai T., Inoue Y., Seki M., Sakakibara H., Sugimoto K. (2011). The AP2/ERF transcription factor WIND1 controls cell dedifferentiation in *Arabidopsis*. Curr. Biol..

[21] Pulianmackal A.J., Kareem A.V.K., Durgaprasad K., Trivedi Z.B., Prasad K. (2014). Competence and regulatory interactions during regeneration in plants. Front. Plant Sci..

[22] Liu J., Sheng L., Xu Y., Li J., Yang Z., Huang H., Xu L. (2014). *WOX11* and *12* are involved in the first-step cell fate transition during de novo root organogenesis in *Arabidopsis*. Plant Cell.

[23] Lopes F.L., Galvan-Ampudia C., Landrein B. (2021). *WUSCHEL* in the shoot apical meristem: Old player, new tricks. J. Exp. Bot..

[24] Meng W.J., Cheng Z.J., Sang Y.L., Zhang M.M., Rong X.F., Wang Z.W., Tang Y.Y., Zhang X.S. (2017). Type-B ARABIDOPSIS RESPONSE REGULATORs specify the shoot stem cell niche by dual regulation of *WUSCHEL*. Plant Cell.

[25] Motte H., Vercauteren A., Depuydt S., Landschoot S., Geelen D., Werbrouck S., Goormachtig S., Vuylsteke M., Vereecke D. (2014). Combining linkage and association mapping identifies *RECEPTOR-LIKE PROTEIN KINASE1* as an essential *Arabidopsis* shoot regeneration gene. Proc. Natl. Acad. Sci. USA.

[26] Cheon J., Park S.Y., Schulz B., Choe S. (2010). *Arabidopsis* brassinosteroid biosynthetic mutant *dwarf7-1* exhibits slower rates of cell division and shoot induction. BMC. Plant Biol..

[27] Shin J., Bae S., Seo P.J. (2020). De novo shoot organogenesis during plant regeneration. J. Exp. Bot.

[28] Bidabadi S.S., Jain S.M. (2020). Cellular, molecular, and physiological aspects of in vitro plant regeneration. Plants.

[29] Bao Y., Dharmawardhana P., Mockler T.C., Strauss S.H. (2009). Genome scale transcriptome analysis of shoot organogenesis in *Populus*. BMC. Plant Biol..

[30] Wan Q., Zhai N., Xie D., Liu W., Xu L. (2023). *WOX11*: The founder of plant organ regeneration. Cell Regen..

[31] Liu B., Zhang J., Yang Z., Matsui A., Seki M., Li S., Yan X., Kohnen M.V., Gu L., Prasad K. (2018). *PtWOX11* acts as master regulator conducting the expression of key transcription factors to induce de novo shoot organogenesis in poplar. Plant Mol. Biol..

[32] Wasternack C., Song S. (2017). Jasmonates: Biosynthesis, metabolism, and signaling by proteins activating and repressing transcription. J. Exp. Bot..

[33] Liu H., Wang C., Li C., Zhao Z., Wei L., Liu Z., Hu D., Liao W. (2022). Nitric oxide is involved in hydrogen sulfide-induced adventitious rooting in tomato (*Solanum lycopersicum*). Funct. Plant Biol..

[34] Nisler J. (2024). Beyond expectations: The development and biological activity of cytokinin oxidase/dehydrogenase inhibitors. Biochem. Soc. Trans..

[35] Wang B., Bian B., Wang C., Li C., Fang H., Zhang J., Huang D., Huo J., Liao W. (2019). Hydrogen gas promotes the adventitious rooting in cucumber under cadmium stress. PLoS ONE.

[36] Liu R., Yang G., Wu Y., Rao H., Li X., Li M., Qian P. (2015). Effects of light intensity on associated enzyme activity and gene expression during callus formation of *Vitis vinifera*. Chin. J. Biotechnol..

[37] Mok D.W.S., Mok M.C. (2001). Cytokinin metabolism and action. Annu. Rev. Plant Physiol. Plant Mol. Biol..

[38] Iwase A., Harashima H., Ikeuchi M., Rymen B., Ohnuma M., Komaki S., Morohashi K., Kurata T., Nakata M., Ohme-Takagi M. (2017). WIND1 promotes shoot regeneration through transcriptional activation of *ENHANCER OF SHOOT REGENERATION1* in *Arabidopsis*. Plant Cell.

[39] Ikeuchi M., Sugimoto K., Iwase A. (2013). Plant callus: Mechanisms of induction and repression. Plant Cell.

[40] De Marco M.A., Curatti L., Martínez-Nol G.M.A. (2024). High auxin disrupts expression of cell-cycle genes, arrests cell division and promotes accumulation of starch in *Chlamydomonas reinhardtii*. Algal Res..

[41] Gordon S.P., Heisler M.G., Reddy G.V., Ohno C., Das P., Meyerowitz E.M. (2007). Pattern formation during de novo assembly of the *Arabidopsis* shoot meristem. Development.

[42] Zhai N., Xu L. (2021). Pluripotency acquisition in the middle cell layer of callus is required for organ regeneration. Nat. Plants.

[43] Zhang P., Wang Y., Wang J., Li G., Li S., Ma J., Peng X., Yin J., Liu Y., Zhu Y. (2023). Transcriptomic and physiological analyses reveal changes in secondary metabolite and endogenous hormone in ginger (*Zingiber officinale* Rosc.) in response to postharvest chilling stress. Plant Physiol. Biochem..

[44] Nicolas A., Laufs P. (2022). Meristem initiation and de novo stem cell formation. Front. Plant Sci..

[45] Ikeuchi M., Iwase A., Rymen B., Lambolez A., Kojima M., Takebayashi Y., Heyman J., Watanabe S., Seo M., De Veylder L. (2017). Wounding triggers callus formation via dynamic hormonal and transcriptional changes. Plant Physiol..

[46] Lee K., Seo P.J. (2017). High-temperature promotion of callus formation requires the BIN2-ARF-LBD axis in *Arabidopsis*. Planta.

[47] Yin J., Tian J., Li G., Zhu Y., Zhou X., He Y., Nie P., Su Y., Zhong Q., Chen Z. (2020). Carbohydrate, phytohormone, and associated transcriptome changes during storage root formation in alligatorweed (*Alternanthera philoxeroides*). Weed Sci..

[48] Hong L., Fletcher J.C. (2023). Stem cells: Engines of plant growth and development. Int. J. Mol. Sci..

[49] Zhao X., Song J., Zeng Q., Ma Y., Fang H., Yang L., Deng B., Liu J., Fang J., Zuo L. (2021). Auxin and cytokinin mediated regulation involved in vitro organogenesis of papaya. J. Plant Physiol..

[50] Lombardi-Crestana S., da Silva Azevedo M., e Silva G.F., Pino L.E., Appezzato-da-Glória B., Figueira A., Nogueira F.T., Peres L.E. (2012). The tomato (*Solanum lycopersicum* cv. Micro-Tom) natural genetic variation Rg1 and the DELLA mutant procera control the competence necessary to form adventitious roots and shoots. J. Exp. Bot..

[51] Chen L., Tong J., Xiao L., Ruan Y., Liu J., Zeng M., Huang H., Wang J.W., Xu L. (2016). *YUCCA*-mediated auxin biogenesis is required for cell fate transition occurring during de novo root organogenesis in *Arabidopsis*. J. Exp. Bot..

[52] Sugimoto K., Jiao Y., Meyerowitz E.M. (2010). *Arabidopsis* regeneration from multiple tissues occurs via a root development pathway. Dev. Cell.

[53] Zhai N., Pan X., Zeng M., Xu L. (2023). Developmental trajectory of pluripotent stem cell establishment in *Arabidopsis* callus guided by a quiescent center-related gene network. Development.

[54] Hu X., Xu L. (2016). Transcription Factors *WOX11/12* directly activate *WOX5/7* to promote root primordia initiation and organogenesis. Plant Physiol..

[55] Guarneri N., Willig J.J., Willemsen V., Goverse A., Sterken M.G., Nibbering P., Lozano Torres J.L., Smant G. (2024). *WOX11*-mediated cell size control in *Arabidopsis* attenuates growth and fecundity of endoparasitic cyst nematodes. Plant J..

[56] Nakamura S., Kinoshita A., Koga H., Tsukaya H. (2024). Expression analyses of *CUP-SHAPED COTYLEDON* and *SHOOT MERISTEMLESS* in the one-leaf plant *Monophyllaea glabra* reveal neoteny evolution of shoot meristem. Sci. Rep..

[57] Sang Y.L., Cheng Z.J., Zhang X.S. (2018). Plant stem cells and de novo organogenesis. New. Phytol..

[58] Ikeuchi M., Favero D.S., Sakamoto Y., Iwase A., Coleman D., Rymen B., Sugimoto K. (2019). Molecular mechanisms of plant regeneration. Annu. Rev. Plant Biol..

[59] Negin B., Shemer O., Sorek Y., Eshed Williams L. (2017). Shoot stem cell specification in roots by the *WUSCHEL* transcription factor. PLoS ONE.

[60] Matsuo N., Makino M., Banno H. (2011). *Arabidopsis ENHANCER OF SHOOT REGENERATION (ESR)1* and *ESR2* regulate in vitro shoot regeneration and their expressions are differentially regulated. Plant Sci..

[61] Yan T., Hou Q., Wei X., Qi Y., Pu A., Wu S., An X., Wan X. (2023). Promoting genotype-independent plant transformation by manipulating developmental regulatory genes and/or using nanoparticles. Plant Cell Rep..

[62] Kareem A., Durgaprasad K., Sugimoto K., Du Y., Pulianmackal A.J., Trivedi Z.B., Abhayadev P.V., Pinon V., Meyerowitz E.M., Scheres B. (2015). *PLETHORA* genes control regeneration by a two-step mechanism. Curr. Biol..

[63] Motte H., Vereecke D., Geelen D., Werbrouck S. (2014). The molecular path to in vitro shoot regeneration. Biotechnol. Adv.

[64] Liu Y.J., Wang X.R., Zeng Q.Y. (2019). De novo assembly of white poplar genome and genetic diversity of white poplar population in Irtysh River basin in China. Sci. China. Life. Sci..

[65] Zhao H.Y., Lu S.F. (2001). Chao, R.T. Studies on tissue culture and gene engineering of *Poplar*. Chin. Bull. Bot..

