# Supplementary material for: A Comprehensive Analysis on the Regulatory Network Underlying Callus Induction and Adventitious Organogenesis Process in Stem of Populus Alba L
Source: Int J Mol Sci. 2025 Apr 25;26(9):4087. doi: 10.3390/ijms26094087 (PMC12071542; doi:10.3390/ijms26094087)
Supplement: Supplementary file 1 [file ijms-26-04087-s001.zip › ijms-3492110 - SupFigs.pdf]

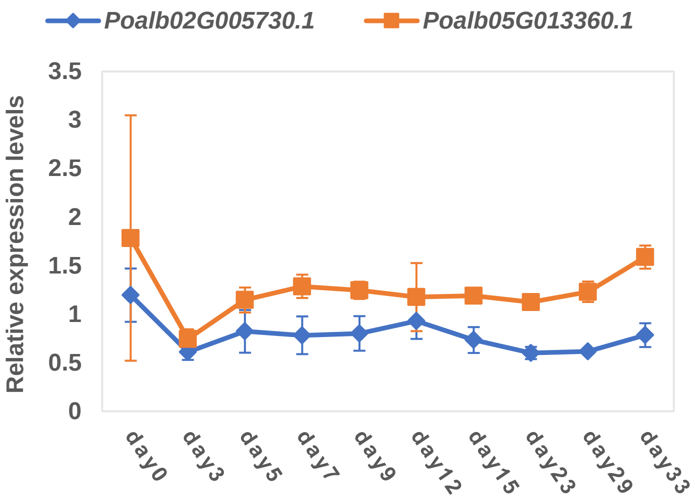

Figure S1. Expression levels of *Arabidopsis WIND1* homologs in *P. alba*. The expression of *WIND* is regarded as markers of wound-response pathways. Their expression profiles were assayed by RT-qPCR to distinguish wound-responsive and hormone-induced pathways. The expression values were normalized by the value of day 0. Data are shown as mean ± standard deviation (SD) for three independent biological replicates.
